# The Incidence and Risk Factors of Medial and Inferior Orbital Wall Fractures in Korea: A Nationwide Cohort Study

**DOI:** 10.3390/jcm11092306

**Published:** 2022-04-21

**Authors:** Eun Hye Jung, Min Joung Lee, Bum-Joo Cho

**Affiliations:** 1 Department of Ophthalmology, Nowon Eulji Medical Center, Eulji University, Seoul 01830, Korea; halogenlove@naver.com; 2 Department of Ophthalmology, Hallym University Sacred Heart Hospital, Hallym University College of Medicine, Anyang 14068, Korea; minjounglee77@gmail.com

**Keywords:** orbital fracture, orbital wall, blowout fracture, incidence

## Abstract

Purpose: We aimed to investigate orbital wall fracture incidence and risk factors in the general Korean population. Method: The Korea National Health Insurance Service–National Sample Cohort dataset was analyzed to find subjects with an orbital wall fracture between 2011 and 2015 (based on the diagnosis code) and to identify incident cases involving a preceding disease-free period of 8 years. The incidence of orbital wall fracture in the general population was estimated, and the type of orbital wall fracture was categorized. Sociodemographic risk factors were also examined using Cox regression analysis. Results: Among 1,080,309 cohort subjects, 2415 individuals with newly diagnosed orbital wall fractures were identified. The overall incidence of orbital wall fractures was estimated as 46.19 (95% CI: 44.37–48.06) per 100,000 person-years. The incidence was high at 10–29 and 80+ years old and showed a male predominance with an average male-to-female ratio of 3.33. The most common type was isolated inferior orbital wall fracture (59.4%), followed by isolated medial orbital wall fracture (23.7%), combination fracture (15.0%), and naso-orbito-ethmoid fracture (1.5%). Of the fracture patients, 648 subjects (26.8%) underwent orbital wall fracture repair surgeries. Male sex, rural residence, and low income were associated with an increased risk of orbital wall fractures. Conclusions: The incidence of orbital wall fractures in Korea varied according to age groups and was positively associated with male sex, rural residency, and low economic income. The most common fracture type was an isolated inferior orbital wall fracture.

## 1. Introduction

An orbital wall fracture is a common consequence of blunt trauma around the eye and is frequently encountered in visits associated with periorbital trauma [1,2,3]. The bony walls of the orbit are very thin and susceptible to injury, so a momentary increase in intra-orbital pressure due to the periorbital injury can fracture bony orbital walls [3,4]. The main signs and symptoms of orbital wall fracture include periorbital ecchymosis, ocular motility restriction, diplopia, and enophthalmos [5]. Many subspecialists, including ophthalmologists, otolaryngologists, maxillofacial specialists, neurosurgeons, and even plastic surgeons, are involved in evaluating and treating orbital wall fractures [1].

Thus far, many clinical studies have reported the etiologies and clinical presentations of orbital wall fractures at a single tertiary center or single department [3,4,5], whereas nationwide epidemiologic studies on orbital wall fractures are still limited [6]. The incidence of orbital wall fractures has been presented as part of facial fractures in only a few studies [7,8] or examined for the orbital floor fracture type in the general population [2]. The specific incidence rates of medial and inferior orbital wall fractures have rarely been reported in a large population. Further epidemiological studies for orbital wall fractures might help to estimate the scale of the disease and measure the socioeconomic burden, and thus to establish appropriate prevention measures and provide evidence for policy making [8].

South Korea is an appropriate country for conducting epidemiological studies, owing to its unique health insurance system covering the entire public [9]. The Korea National Health Insurance Service–National Sample Cohort (NHIS-NSC) database is being provided to represent the general Korean population, or the entire population of Korea. Therefore, in this study, we aimed to investigate orbital wall fracture incidence and demographic information in the general Korean population using the NHIS-NSC database.

## 2. Methods

### 2.1. Dataset and Study Population

The NHIS of Korea has covered about 97% of people in Korea since 1963, leaving the remaining 3% insured by the Medical Aid program [9,10]. All claims in the NHIS have been recorded in a centralized database and include information regarding patient demographics, diagnostic codes, procedure types, drug prescriptions, and related costs [9,10]. The NHIS-NSC database was constructed to represent the general Korean population for research purposes and consisted of approximately 1 million participants (2.2% of the Korean population) selected by random sampling stratified for age, sex, income, residential area, and income [9,11]. Regarding diagnostic coding, the Korean Classification of Diseases (KCD) system, which is based on the International Classification of Diseases 10th Edition (ICD-10), was adopted in the NHIS-NSC system [9]. Details of the construction and analysis method of the NHIS-NSC are described elsewhere [9]. We used the second version of the NHIS-NSC dataset, which covers the period from 2002 to 2015 and was released in 2017. Ethical review and approval were waived for this study by the Institutional Review Board of Hallym University Medical Center (IRB No. 2020-04-030) and this study complied with the tenets of the Declaration of Helsinki.

### 2.2. Definition and Classification of Orbital Wall Fractures

Orbital wall fracture was defined as a diagnosis of orbital wall fracture according to KCD codes. Patients with orbital wall fractures were identified using claims with the KCD code for fracture of orbital floor (S02.3), LeFort 2 (S02.43), LeFort 3 (S02.45, S02.46), naso-orbito-ethmoid (S02.72), and medial wall of orbit (S02.84) from 2011 to 2015. The superior and lateral orbital wall fractures are not specifically coded in the NHIS-NSC database and thus were excluded in this study. The sixth edition of the KCD system, which corresponds to the ICD-10, was revised in January 2011, so a database with lower hierarchical level and specific diagnostic codes to the second decimal place has been available since then. Therefore, we identified patients with orbital wall fractures after this date.

All cases were classified or typed into medial orbital wall fracture, inferior orbital wall fracture, Le Fort fracture, or naso-orbito-ethmoidal fracture according to KCD codes. If multiple diagnostic codes were recorded, the subject’s type was categorized as a combination fracture [4]. An incident date was defined based on the earliest orbital wall fracture diagnosis for each subject. Patients diagnosed with orbital wall fracture between 2002 and 2010 were excluded as a preceding 8-year disease-free period [11]. We obtained annual incidence rates according to age group (5-year intervals), sex, and year. The annual incidence was estimated using the number of patients who qualified for NHIS-in each year [12].

Surgery for orbital wall fractures, defined as surgeries performed after the diagnosis of orbital wall fractures during the follow-up period, was identified using the Korean Electronic Data Interchange codes for reconstruction of orbital wall fracture (S521).

### 2.3. Sociodemographic Risk Factors of Orbital Wall Fractures

In the NHIS-NSC database of Korea, sociodemographic factors are provided as eligibility data including age, sex, residential area, and income, and are updated annually [9,12]. Factors potentially associated with orbital wall fracture were investigated to identify risk factors. Other factors such as systemic conditions were unavailable and thus excluded from this study. We classified ages into 10-year intervals and residence into 3 administrative districts (Seoul, Korea’s metropolitan capital city; metropolitan cities; and provinces of Korea) to reflect characteristics of urban and rural areas [13]. In previous study, we analyzed income levels using health insurance premiums as a proxy indicator of the estimated income level. The income levels for determining health insurance premiums were classified into 1 of 10 groups ranging from 1 to 10 [13]. In this study, income levels were grouped into low-income (first to third premium quantiles and medical aid beneficiaries), medium-income (fourth to seventh premium quantiles), and high-income groups (eighth to tenth premium quantiles).

### 2.4. Statistical Analysis

The incidence was estimated with a 95% confidence interval (CI) based on a Poisson distribution. Factors associated with incidence rates over time were evaluated using the multivariable Cox proportional hazards regression model. A two-sided *p*-value of <0.05 was considered statistically significant for risk factor evaluation. All statistical analyses were performed using SAS Enterprise Guide version 7.1 (SAS Institute, Cary, NC, USA) and R version 3.4.3 (The R Foundation for Statistical Computing, Vienna, Austria).

## 3. Results

### 3.1. Incidence of Orbital Wall Fractures

A total of 1,085,209 subjects were included in the cohort from 2011 to 2015. A total of 4900 subjects with a history of orbital wall fractures from 2002 to 2010 were excluded for incidence estimation. Among 1,080,309 subjects, 2415 individuals were newly diagnosed with orbital wall fractures at least once from 2011 to 2015. Among them, 1855 (76.8%) were male, and 560 (23.2%) were female.

**Figure 1:**
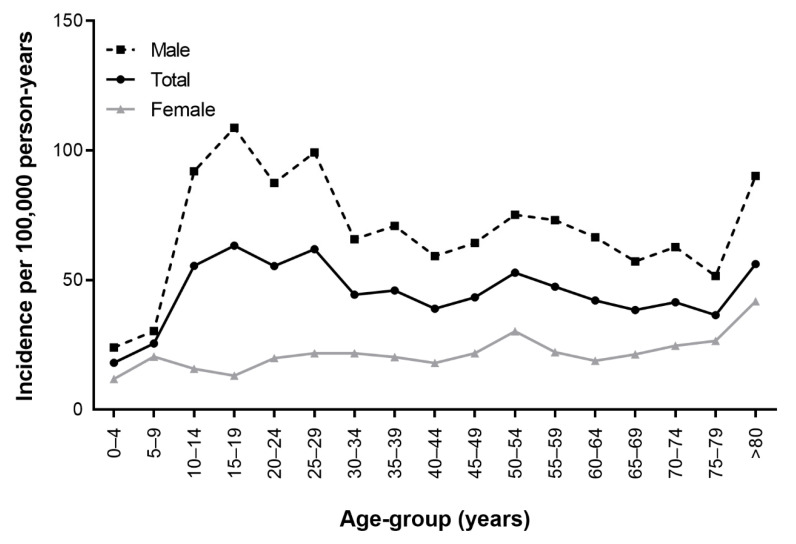
Incidence of orbital wall fractures by age group.

**Figure 2:**
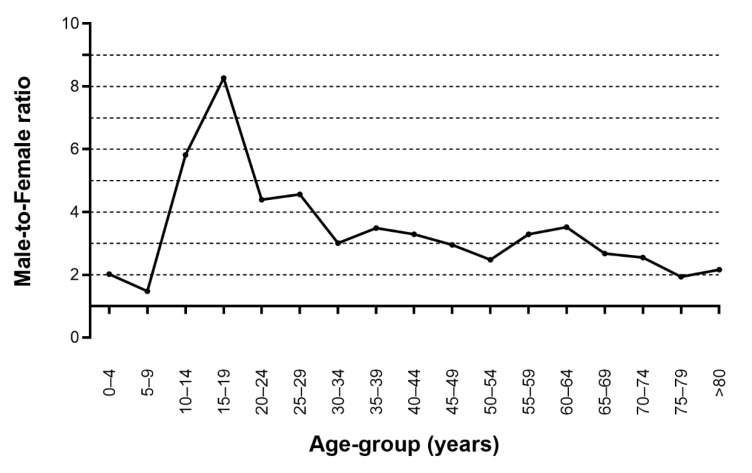
Male-to-female ratio for the incidence of orbital wall fractures.

Table 1 and Figure 1 show the incidence of orbital wall fractures according to age-group and sex. The estimated overall incidence of orbital wall fractures in the cohort was 46.19 (95% CI: 44.37–48.06) per 100,000 person years. The incidence of orbital wall fractures was 71.12 (95% CI: 67.93–74.40) per 100,000 person years for males and 21.37 (95% CI: 19.65–23.19) per 100,000 person years for females. The incidence of orbital wall fractures was high at ages 10–29, 50–54, and over 80 in males and in the entire cohort. In females, the incidence of orbital wall fractures was highest at over 80 years old. Figure 2 shows the male-to-female ratio of the incidence of orbital wall fractures according to age group. The incidence of orbital wall fractures was higher in males than in females, and the average male-to-female incidence ratio was 3.33. Male preponderance was highest in subjects aged 15–19 and was higher in the groups of subjects aged 10–29.

The annual incidence of orbital wall fractures showed a slight decrease in the study period, from 49.87 (95% CI: 45.70–54.29) in 2011 to 44.70 (95% CI: 40.78–48.87) in 2015 (Table 2).

### 3.2. Types of Orbital Wall Fractures

The types of orbital wall fractures are presented in Table 3. Of 2415 subjects, inferior orbital wall fracture was the most common (*n* = 1435, 59.4%), followed by the medial orbital wall (*n* = 572, 23.7%), a combination (*n* = 363, 15.0%), naso-orbito-ethmoid (*n* = 37, 1.5%), and Le Fort (*n* = 8, 0.3%). As 363 subjects had multiple diagnostic codes, 2788 orbital wall fractures were diagnosed in 2415 subjects. Of 363 subjects with combination fractures, medial and inferior orbital wall fracture was most common (*n* = 333, 91.7%); followed by naso-orbito-ethmoid and inferior orbital wall (*n* = 9, 2.5%), medial, inferior orbital wall, and naso-orbito-ethmoid (*n* = 6, 1.7%); Le Fort and inferior orbital wall (*n* = 6, 1.7%); medial, inferior orbital wall, and Le Fort (*n* = 4, 1.1%); medial orbital wall and Le Fort (*n* = 3, 0.8%); and medial orbital wall and naso-orbito-ethmoid (*n* = 2, 0.6%).

Of the 2415 subjects diagnosed with orbital wall fractures from 2011 to 2015, 648 subjects underwent 659 orbital wall fractures surgeries (26.8%). The proportion of surgery per patient by fracture type was the highest with combination (*n* = 194, 53.4%), inferior wall (*n* = 358, 24.9%), medial wall (*n* = 95, 16.6%), naso-orbito-ethmoid (*n* = 1, 2.7%), and Le Fort (*n* = 0) fractures.

### 3.3. Sociodemographic Risk Factors of Orbital Wall Fractures

Table 4 shows the hazard ratio (HR) for orbital wall fractures during the follow-up period using the multivariable Cox proportional hazard regression model. In terms of age groups, subjects 10–19, 20–29, 50–59, and >80 years old were significantly more likely to have orbital wall fractures compared with subjects 40–49 years old (*p* < 0.001, *p* < 0.001, *p* = 0.005, and *p* < 0.001, respectively). The 0–9 age group was less likely to have orbital wall fractures than other age groups (*p* < 0.001).

Males had a significantly high risk for orbital wall fracture (OR 3.38, 95% CI 3.07–3.72, *p* < 0.001). In terms of the area of residence, rural province living was a risk factor for orbital wall fractures (OR: 1.17, 95% CI: 1.05–1.30, *p* = 0.005). A lower economic income level was also significantly associated with an increased risk of orbital wall fractures, compared to a high income level. (low, OR: 1.31, 95% CI: 1.18–1.45, *p* < 0.001; medium, OR: 1.15, 95% CI; 1.05–1.26, *p* = 0.004).

## 4. Discussion

In this study, we investigated the incidence, anatomical location, and demographic information of orbital wall fractures in South Korea from 2011 to 2015. We found that the incidence of orbital wall fractures was estimated at 46.19 per 100,000 person-years. Surgeries were performed in 26.8% of diagnosed subjects with orbital wall fractures. Orbital wall fractures were most likely to occur in young men aged 10–29 and elderly groups aged over 80 years old, and the most common fracture site was the inferior orbital wall. Male sex, a relatively rural residence, and lower income were associated with an increased risk of orbital wall fractures.

Orbital wall fracture is a quite common status, but the incidence and prevalence rates of orbital wall fracture are not clearly elucidated. Notably, the global incidence rate of facial fracture was 98 per 100,000 in 2017 [8], and the incidence of orbital floor fracture was 11.3 per 100,000 people in 2017 in the United States [2]. In the present study, the overall incidence of medial and inferior orbital wall fractures (46.19 per 100,000 person-years) was somewhere between that of facial bone fracture and that of orbital floor fracture. Considering approximately 60% of the overall fracture was inferior wall fracture, the estimated incidence for orbital wall fractures in this study might be higher than that in the United States [2]. This difference is not fully explained, but the easy access to medical services in Korea might be one reason.

Interestingly, a few studies have reported the increasing prevalence or incidence of orbital wall fractures [2,6,14]. The incidence of orbital floor fracture increased from 7.7 to 11.3 per 100,000 people in the United States [2]. However, Kwon et al. reported that the incidence of major ocular trauma decreased steadily from 2010 to 2018 in Korea [15]. Park et al. also reported that the incidence of facial fractures per 100,000 people had decreased from 212 in 2011 to 171 in 2016 [7]. Our results also showed a decreasing trend in the incidence rates. Although the incidence of orbital fractures is decreasing, considering that Korea is an aging society, preventing orbital wall fractures in the elderly might be necessary to economize national health costs [7].

The etiology of orbital wall fracture includes physical assault, blunt blows, falls, motor vehicle accidents, sports, work-related injuries, and several other accidents [2,4]. Iftikhar et al. reported that the most common cause of orbital floor fractures was assault (43%), which was most frequent in young adults (65%), along with falls (26%), most frequent in patients over 65 years old (86%) [2]. Other studies reported that orbital wall fractures occur more frequently in male patients 20–30 years old [4,5]. As expected, we found a male predominance, with the highest number of orbital fractures seen in those in their second or third decade of life, and this is consistent with other previous epidemiology studies [4,5,16,17]. This could be explained by the possibility of a predominance of the male population in road traffic activities, aggressive behavior such as physical assault, and higher male employment in occupations with more significant risks of trauma leading to orbital fractures when compared with female patients [4]. However, in terms of the incidence rate, we found that the subjects over 80 years old demonstrated as high a rate of injury as the young male group. Previous studies reported that most patients with orbital wall fractures were seen in 20–30-year-old men, and older patients comprised less than 10% [4,5,16]. Falls in older people might be associated with ocular injuries, such as orbital wall fractures, which are common and easy to miss in the population [18]. Thus, physicians should consider this potential oversight and perform a detailed assessment of elderly patients.

An orbital fracture can involve any or all of the surrounding bony parts, but the floor and the medial wall are more commonly involved due to the thin thickness of the bone [4]. Studies reported that the location of orbital fractures varied by race, and the most common site for orbital fractures is the orbital floor in Caucasian people, whereas the medial wall is most common in Afro-Caribbean people [19,20]. Previous anatomical studies have reported the possibilities of racial variations in the shape of the orbit or the partition of the ethmoid sinus [21,22]. An orbital floor fracture is the most common location, and this might be due to anatomy, with the orbital floor being the thinnest and weakest area of the orbit in Europe and the United States [17,23]. There are controversial reports about the most common anatomical location involved in orbital wall fracture in Asians [19]. Studies in a single institution have reported that a fracture is more likely to occur in the orbital floor in Asians [14,19]. However, a number of studies in Asian tertiary centers have demonstrated that fractures tend to occur more commonly in the medial wall than in the orbital floor [3,4,5,16,20,24]. One study reported orbital wall fractures in the general Korean population, and they reported that an inferior orbital wall fracture was the most common [7]. Our study also found that inferior orbital wall fracture was the most commonly affected site in Korea. Further anatomic studies using computed tomography (CT) scans are required to examine the Asian orbit and better characterize these fracture patterns. We found that patients with combined orbital wall fractures have a higher surgical rate than other orbital wall fractures. The proportion of surgery was 26.8% in our study, similar to the prevalence of 20.7 to 25% in other retrospective review studies [6,25].

Our study has several limitations. First, the diagnosis of orbital wall fractures relied on diagnostic codes from the NHIS claims data. The diagnoses were not confirmed by a detailed review of the medical records, including CT images, because they are not provided in the NHIS claim database. Our study only included patients with an exact diagnosis of orbital fractures, excluding patients diagnosed with other facial fractures codes including fractures of other and unspecified skull and facial bones. Additionally, we did not distinguish between isolated pure orbital fractures and complex fractures, including other facial bone fractures. Thus, there is the possibility of missing data and errors. In addition, the incidence could have been underestimated because subjects who had not received medical care were not included in the NHIS-NSC database.

In conclusion, the present study investigated the incidence of medial and inferior orbital fractures in the general population, which is not well-known, and estimated the incidence rate in Korea as 46.19 per 100,000 person-years. The inferior orbital wall fracture was the most common fracture site. Male sex, specific age groups, low socioeconomic status, and rural geographic residence influenced the incidence rate of orbital wall fractures. Young males and elderly people were at a higher risk of orbital wall fractures. Our study provides an insight into the current trends in the demographics of orbital wall fractures that will help guide prevention strategies, treatment, and appropriate resource allocation. To reveal the association mechanism between these risk factors and orbital wall fractures, further clinical studies would be required.

## Figures and Tables

**Table 1:** Number of patients with incident orbital wall fractures and estimated incidence rate (per 100,000 person years) of orbital wall fractures in South Korea.

Age (Years)	Total				Male				Female				MF Ratio	Surgery
	Person Years	N	Incidence	95% CI	Person Years	N	Incidence	95% CI	Person Years	N	Incidence	95% CI		N
0–4	242,563	44	18.14	13.29, 24.04	124,724	30	24.05	16.44, 33.72	117,839	14	11.88	6.69, 19.23	2.02	2
5–9	242,428	62	25.57	19.73, 32.48	125,175	38	30.36	21.70, 41.06	117,253	24	20.47	13.33, 29.78	1.48	8
10–14	290,061	161	55.51	47.37, 64.53	151,045	139	92.03	77.56, 108.18	139,016	22	15.83	10.10, 23.39	5.82	31
15–19	351,996	223	63.35	55.40, 72.04	184,809	201	108.76	94.41, 124.50	167,186	22	13.16	8.40, 19.45	8.27	74
20–24	349,817	194	55.46	48.02, 63.63	184,148	161	87.43	74.61, 101.64	165,669	33	19.92	13.87, 27.51	4.39	79
25–29	334,257	207	61.93	53.87, 70.75	173,322	172	99.24	85.14, 114.82	160,935	35	21.75	15.32, 29.77	4.56	77
30–34	403,632	179	44.35	38.16, 51.16	206,657	136	65.81	55.36, 77.50	196,976	43	21.83	15.94, 29.02	3.01	67
35–39	408,812	188	45.99	39.72, 52.88	207,303	147	70.91	60.06, 83.00	201,509	41	20.35	14.74, 27.23	3.49	72
40–44	461,802	180	38.98	33.56, 44.95	234,447	139	59.29	49.97, 69.70	227,355	41	18.03	13.06, 24.13	3.29	56
45–49	435,864	189	43.36	37.47, 49.84	220,613	142	64.37	54.35, 75.54	215,252	47	21.83	16.17, 28.69	2.95	47
50–54	438,524	232	52.9	46.39, 60.01	220,699	166	75.22	64.35, 87.24	217,826	66	30.3	23.57, 38.21	2.48	54
55–59	366,242	174	47.51	40.80, 54.92	181,771	133	73.17	61.43, 86.32	184,471	41	22.23	16.10, 29.74	3.29	34
60–64	258,453	109	42.17	34.74, 50.59	126,238	84	66.54	53.31, 81.80	132,215	25	18.91	12.43, 27.32	3.52	24
65–69	205,457	79	38.45	30.58, 47.56	97,855	56	57.23	43.52, 73.55	107,602	23	21.38	13.79, 31.34	2.68	10
70–74	180,909	75	41.46	32.77, 51.56	79,602	50	62.81	46.97, 81.87	101,307	25	24.68	16.22, 35.65	2.55	9
75–79	131,376	48	36.54	27.15, 47.87	52,304	27	51.62	34.52, 73.61	79,072	21	26.56	16.76, 39.59	1.94	2
>80	126,253	71	56.24	44.15, 70.35	37,710	34	90.16	63.15, 123.95	88,543	37	41.79	29.73, 56.74	2.16	2
Overall	5,228,446	2415	46.19	44.37, 48.06	2,608,422	1855	71.12	67.93, 74.40	2,620,026	560	21.37	19.65, 23.19	3.33	648

**Table 2:** Annual incidence of orbital wall fractures in the general Korean population from 2011 to 2015.

Year	Incidence			
	Person Years	No.	Incidence	95% CI
2011	1,038,607	518	49.87	45.70, 54.29
2012	1,045,182	493	47.17	43.13, 51.46
2013	1,045,101	492	47.08	43.04, 51.36
2014	1,048,204	442	42.17	38.36, 46.22
2015	1,051,352	470	44.70	40.78, 48.87

**Table 3:** The site of the orbital wall fractures in this study.

Fracture Sites	N (%)
Inferior orbital wall	1435 (59.4)
Medial orbital wall	572 (23.7)
Le Fort II or III	8 (0.3)
Naso-orbito-ethmoid	37 (1.5)
Combination	363 (15.0)
Total	2415

**Table 4:** Factors associated with orbital wall fractures based on multivariable Cox regression analysis.

	HR	95% CI	*p*-Value
Age Group(Year)			
0–9	0.52	0.42, 0.65	<0.001
10–19	1.43	1.24, 1.65	<0.001
20–29	1.37	1.19, 1.58	<0.001
30–39	1.1	0.95, 1.27	0.198
40–49	1 (ref)		
50–59	1.22	1.06, 1.41	0.005
60–69	1.01	0.85, 1.21	0.9
70–79	1.06	0.86, 1.30	0.596
>80	1.75	1.35, 2.26	<0.001
Sex			
Male	3.38	3.07, 3.72	<0.001
Female	1 (ref)		
Residence			
Province	1.17	1.05, 1.30	0.005
Metropolitan city	1.1	0.97, 1.24	0.138
Seoul	1 (ref)		
Income			
Low	1.31	1.18, 1.45	<0.001
Middle	1.15	1.05, 1.26	0.004
High	1 (ref)		
